# Gastric Pancreatic Heterotopia and Bronchoesophageal Fistula in a Patient With Systemic Lupus Erythematosus and Antiphospholipid Syndrome: A Case Report

**DOI:** 10.7759/cureus.117879

**Published:** 2026-10-13

**Authors:** Anhar S Alqahtani, Ahmed M Alasmari, Asma S Aldahmashi, Hayam S Almadani

**Affiliations:** 1 General Surgery, Asir Central Hospital, Abha, SAU; 2 Bariatric and Upper Gastrointestinal Surgery, Asir Central Hospital, Abha, SAU

**Keywords:** antiphospholipid syndrome, bronchoesophageal fistula, gastric pancreatic heterotopia, gastrointestinal bleeding, systemic lupus erythematosus

## Abstract

A 23-year-old woman with documented systemic lupus erythematosus (SLE) and antiphospholipid syndrome (APS) and a recent pulmonary embolism requiring anticoagulation presented with acute hematemesis. Upper gastrointestinal endoscopy revealed a small mid-esophageal opening with mucus and air passage and an ulcerated gastric subepithelial lesion, without an identifiable active bleeding source. Chest computed tomography with oral contrast demonstrated a left bronchoesophageal fistula (BEF). Endoscopic ultrasonography showed a 20.8 × 15.4 mm hypoechoic lesion arising from the fourth sonographic layer of the gastric wall, corresponding to the muscularis propria, in the fundus/body region. The lesion was ulcerated and endophytic, raising suspicion for a gastrointestinal stromal tumor (GIST). EUS-guided tissue acquisition was deferred because of anticoagulation, and laparoscopic resection was performed. Histopathological examination revealed Heinrich type I gastric pancreatic heterotopia (GPH) involving the submucosa and muscularis propria, with pancreatic acini, ducts, and islet cells, accompanied by focal pancreatitis and chronic active gastritis. No malignancy was identified, and the surgical margins were free. Perioperative anticoagulation was managed by temporary interruption of warfarin with therapeutic enoxaparin bridging. The available records did not document definitive fistula-directed treatment or establish whether the BEF subsequently resolved or persisted. Although GPH and BEF coexisted in this patient, no direct anatomical or causal relationship could be established, and the source of hematemesis remained undetermined. This case highlights the diagnostic challenges of concurrent gastric and esophageal abnormalities in a patient with competing thrombotic and bleeding risks and underscores the importance of multimodal imaging, histopathological confirmation, and individualized perioperative anticoagulation management.

## Introduction

Gastric pancreatic heterotopia (GPH), also referred to as ectopic or heterotopic pancreas, is a congenital developmental anomaly characterized by the presence of pancreatic tissue outside the normal pancreas [1]. It is most frequently identified in the upper gastrointestinal tract, particularly the stomach and duodenum, and is often discovered incidentally [1]. Although most lesions are asymptomatic, symptomatic GPH may present with abdominal pain, gastrointestinal bleeding, pancreatitis, or gastric outlet obstruction, and surgical or endoscopic resection is frequently performed in symptomatic cases [2].

The diagnosis of GPH can be challenging because it frequently resembles other gastric subepithelial lesions, particularly gastrointestinal stromal tumors (GISTs) and leiomyomas [3]. Endoscopic ultrasonography may demonstrate a hypoechoic lesion arising from the deeper gastric wall layers, but these findings are not always sufficiently specific to establish the diagnosis [3]. Consequently, histopathological examination remains important, particularly when lesions are symptomatic, ulcerated, enlarging, or radiologically atypical. Reported complications include ulceration, bleeding, pancreatitis, obstruction, and, rarely, fistulization [2,3]. Thus, an ulcerated gastric subepithelial lesion may remain diagnostically indeterminate on endoscopy and endoscopic ultrasonography alone, particularly when tissue acquisition is not feasible.

Bronchoesophageal fistula (BEF) is an uncommon communication between the esophagus and tracheobronchial tree that may result in substantial morbidity. In adults, benign non-iatrogenic BEF has diverse etiologies, including congenital malformations, infections, and idiopathic disease, and diagnosis may require complementary imaging and endoscopic modalities [4]. Notably, a published 1989 case reported gastrobronchial fistula associated with pancreatic heterotopia, demonstrating that fistulization involving ectopic pancreatic tissue, although exceptionally uncommon, has previously been described [5]. However, the coexistence of GPH with a BEF remains unusual, and the anatomical and causal relationship between these two abnormalities is not established.

The diagnostic and therapeutic complexity may be further increased in patients with systemic lupus erythematosus (SLE) and antiphospholipid syndrome (APS), particularly when thrombotic disease necessitates long-term anticoagulation. In this setting, gastrointestinal bleeding requires careful evaluation because bleeding risk and thrombotic risk may coexist, making attribution of bleeding to anticoagulation alone potentially misleading [6,7]. Importantly, the presence of anticoagulation in the present case should be considered a management context rather than evidence that anticoagulation caused or contributed to the gastric lesion or fistula.

The knowledge gap addressed by this case is the diagnostic challenge created by the simultaneous identification of an ulcerated gastric subepithelial lesion and an unexpected esophageal-airway communication in a patient with competing thrombotic and bleeding risks. The purpose of this report is not to propose a causal relationship between GPH and BEF, but to highlight the importance of maintaining a broad differential diagnosis, using multimodal imaging, and obtaining histopathological confirmation when feasible. We report a rare case of GPH occurring in association with BEF in a patient with SLE, APS, and anticoagulation, emphasizing the diagnostic challenge and the multidisciplinary considerations required for management.

## Case presentation

A 23-year-old Saudi woman with recently diagnosed SLE and a documented diagnosis of APS presented with acute hematemesis. Approximately one month before presentation, she had been diagnosed with pulmonary embolism and commenced anticoagulation. Approximately three months before admission, she had sustained a left ankle sprain followed by swelling and pain requiring cast immobilization for approximately one month. She subsequently developed left-leg heaviness and tightness. During evaluation for pulmonary embolism, she was found to have features suggestive of an underlying autoimmune disease, including arthralgia, oral ulcers, hair loss, and cutaneous lesions. Positive antinuclear and antiphospholipid antibodies supported the documented diagnoses of SLE and APS. The diagnosis of APS is reported here as documented in her medical record; repeat antiphospholipid antibody testing to establish persistent laboratory positivity was not available in the records reviewed. Her family history was notable for recurrent deep-vein thrombosis in her mother and SLE in her twin. The major clinical events and diagnostic findings are summarized in Table 1.

**Table 1:** Clinical course and major diagnostic findings Abbreviations: ANA, antinuclear antibody; CT, computed tomography; EUS, endoscopic ultrasonography; GIST, gastrointestinal stromal tumor; LV, left ventricular; PE, pulmonary embolism; SLE, systemic lupus erythematosus; APS, antiphospholipid syndrome

Time point	Key clinical findings	Key investigations	Diagnosis / outcome	Management
Approximately three months before admission	Left ankle sprain requiring approximately one month of cast immobilization, followed by left-leg heaviness and tightness	Not applicable	Recent lower-limb immobilization preceding thromboembolic presentation	Cast immobilization followed by clinical evaluation
03/2025 – Initial presentation	One-week history of chest pain and dyspnea; history of arthralgia, oral ulcers, hair loss, and skin lesions	CT pulmonary angiography demonstrated multiple filling defects involving the distal right main pulmonary artery and right upper-, middle-, and lower-lobar branches, with additional left lower-lobar/subsegmental involvement (Figure 1)	Acute pulmonary embolism; subsequent evaluation documented SLE with antiphospholipid antibody positivity/APS	Enoxaparin and warfarin; rheumatology and hematology evaluation
03/2025 – Systemic evaluation	Bilateral chest and back pain with bilateral pleural effusions, greater on the right	Positive ANA and antiphospholipid antibodies; echocardiography demonstrated preserved left ventricular function with an ejection fraction of 55%	SLE with antiphospholipid antibody positivity/APS, pulmonary embolism, and pleural involvement	Corticosteroids, azathioprine, and continued anticoagulation
04/2025 – Hematemesis and fistula evaluation	Hematemesis and epistaxis while receiving anticoagulation	Upper gastrointestinal endoscopy demonstrated a small mid-esophageal opening with mucus discharge; CT of the chest with oral contrast demonstrated mid-esophageal mural irregularity with extraluminal contrast tracking toward the left main bronchus	Left bronchoesophageal fistula identified on CT; no active bleeding source identified on endoscopy	Multidisciplinary evaluation; chest/cardiothoracic consultation and imaging review recommended; no fistula-directed intervention documented in the available records
04/2025 – Gastric lesion evaluation	Gastric subepithelial lesion identified during endoscopic evaluation	EUS demonstrated a 20.8 × 15.4 mm hypoechoic lesion arising from the fourth sonographic layer (muscularis propria) of the gastric wall, with an endophytic and ulcerated appearance; histopathological examination after resection demonstrated pancreatic tissue within the submucosa and muscularis propria, with focal pancreatitis and no malignancy	Heinrich type I gastric pancreatic heterotopia with focal pancreatitis, initially suspected to be GIST/leiomyoma	Laparoscopic gastric resection; surgical margins were free
2025 – Cutaneous evaluation	Multiple scaly hypopigmented lesions over the extremities	Skin biopsy demonstrated findings consistent with cutaneous lupus erythematosus, favoring discoid lupus erythematosus	Cutaneous lupus erythematosus, favoring discoid lupus erythematosus	Tacrolimus 0.1% ointment; subsequent clinical improvement
2026 – Follow-up	Continued follow-up for SLE and previous pulmonary embolism; clinical improvement documented	Follow-up records documented persistent pulmonary embolism-related changes; CBC and renal function were reported as within normal limits	Ongoing follow-up for SLE and previous pulmonary embolism	Continued multidisciplinary care and anticoagulation; 06/2026: preconception counseling with planned transition from warfarin to therapeutic enoxaparin

At presentation, she was receiving warfarin 6 mg once daily and enoxaparin 80 mg twice daily, in addition to azathioprine 200 mg daily, hydroxychloroquine 400 mg daily, prednisolone 25 mg twice daily, and omeprazole 20 mg daily. She reported sudden-onset hematemesis beginning 1 day before admission, with five episodes of fresh blood, including episodes witnessed in the emergency department. The episodes were preceded by nausea but were not associated with abdominal pain, jaundice, diarrhea, constipation, or rectal bleeding. She also reported mild shortness of breath and headache. Despite the bleeding, she remained hemodynamically stable on initial assessment. The key laboratory and immunological findings are summarized in Table 2. The source of hematemesis was not definitively established, as no active bleeding source was identified on endoscopy. Accordingly, the bleeding was not attributed to the gastric lesion, the bronchoesophageal fistula, or anticoagulation alone.

**Table 2:** Key laboratory and immunological findings Laboratory values represent the key results available in the reviewed medical records. Coagulation results were obtained during anticoagulant therapy and should therefore be interpreted in this context. Antiphospholipid antibody results are reported as documented; repeat testing required to establish persistent positivity was not available in the reviewed records.

Investigation	Parameter	Result	Reference range*	Clinical relevance
Complete blood count	White blood cell count	2.04 × 10³/µL	4.0–10.0 × 10³/µL	Leukopenia
Complete blood count	Hemoglobin	12.3 g/dL	12.0–16.0 g/dL	Within reference range
Complete blood count	Platelet count	185 × 10³/µL	150–400 × 10³/µL	Within reference range
Complete blood count	Mean corpuscular volume	76.1 fL	80–100 fL	Microcytosis
Renal function	Serum creatinine	0.81 mg/dL	0.50–1.10 mg/dL	Preserved renal function
Renal function	24-hour urinary protein	157 mg/day	<150 mg/day	Borderline elevation
Liver function	Alanine aminotransferase	13 U/L	7–35 U/L	Within reference range
Liver function	Aspartate aminotransferase	19 U/L	8–33 U/L	Within reference range
Liver function	Total bilirubin	0.40 mg/dL	0.2–1.2 mg/dL	Within reference range
Liver function	Albumin	4.40 g/dL	3.5–5.0 g/dL	Within reference range
Coagulation profile	Activated partial thromboplastin time	115.2 s	25–35 s	Prolonged; obtained during anticoagulant therapy
Coagulation profile	Prothrombin time	29.2 s	11–14 s	Prolonged; obtained during anticoagulant therapy
Coagulation profile	International normalized ratio	2.36	0.8–1.2	Therapeutic anticoagulation range
Anticardiolipin antibodies	IgG	117.1 U/mL	<15 U/mL	Positive
Anticardiolipin antibodies	IgM	20.7 U/mL	<15 U/mL	Positive
Anti-β2-glycoprotein I antibodies	IgG	117.2 U/mL	<20 U/mL	Positive
Anti-β2-glycoprotein I antibodies	IgM	20.4 U/mL	<20 U/mL	Borderline/positive according to assay cutoff

Because gastrointestinal bleeding in the setting of anticoagulation was initially considered, upper gastrointestinal endoscopy was performed. Esophagogastroduodenoscopy demonstrated a small opening approximately 20 cm from the incisors, with visible air bubbles and mucus discharge, raising suspicion of an abnormal esophageal communication (Figure 1). A gastric subepithelial lesion was also identified. No biopsy was obtained, and no active bleeding source was identified. The remaining gastric and duodenal mucosa was largely unremarkable apart from nodular gastritis. CT of the chest with oral contrast was therefore recommended.

**Figure 1:**
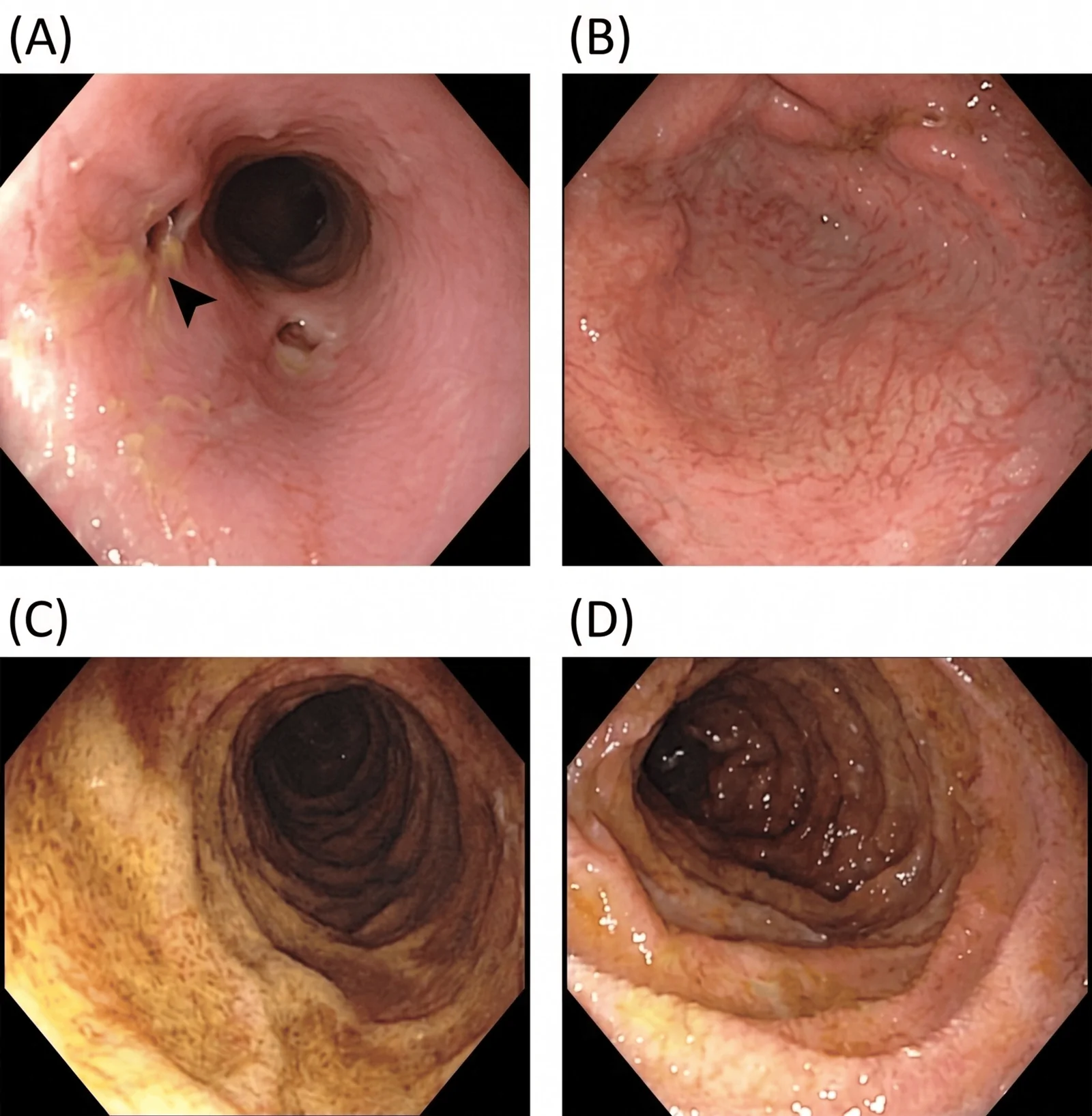
Upper gastrointestinal endoscopic findings demonstrating an abnormal mid-esophageal opening (A) Small abnormal opening in the mid-esophagus with associated mucus discharge and air passage (arrow). (B–D) Representative additional endoscopic views obtained during the examination.

In 04/2025, CT of the chest with oral contrast demonstrated mural irregularity of the mid-esophagus with minimal extraluminal passage of oral contrast toward the left main bronchus, consistent with a left bronchoesophageal fistula. A persistent filling defect within the right main pulmonary artery was also noted, consistent with her recent pulmonary embolism (Figure 2). Following identification of the fistula, review of the imaging and consultation with the chest/cardiothoracic team were recommended. The available records did not document a fistula-directed intervention, such as surgical repair, endoscopic closure, or stent placement. The available follow-up records did not contain definitive repeat imaging establishing whether the fistula subsequently resolved or persisted. The anatomical location of the fistula in the mid-esophagus and the gastric lesion in the fundus/body did not demonstrate direct anatomical continuity between the two abnormalities. Therefore, their coexistence was considered an association rather than evidence of a causal relationship.

**Figure 2:**
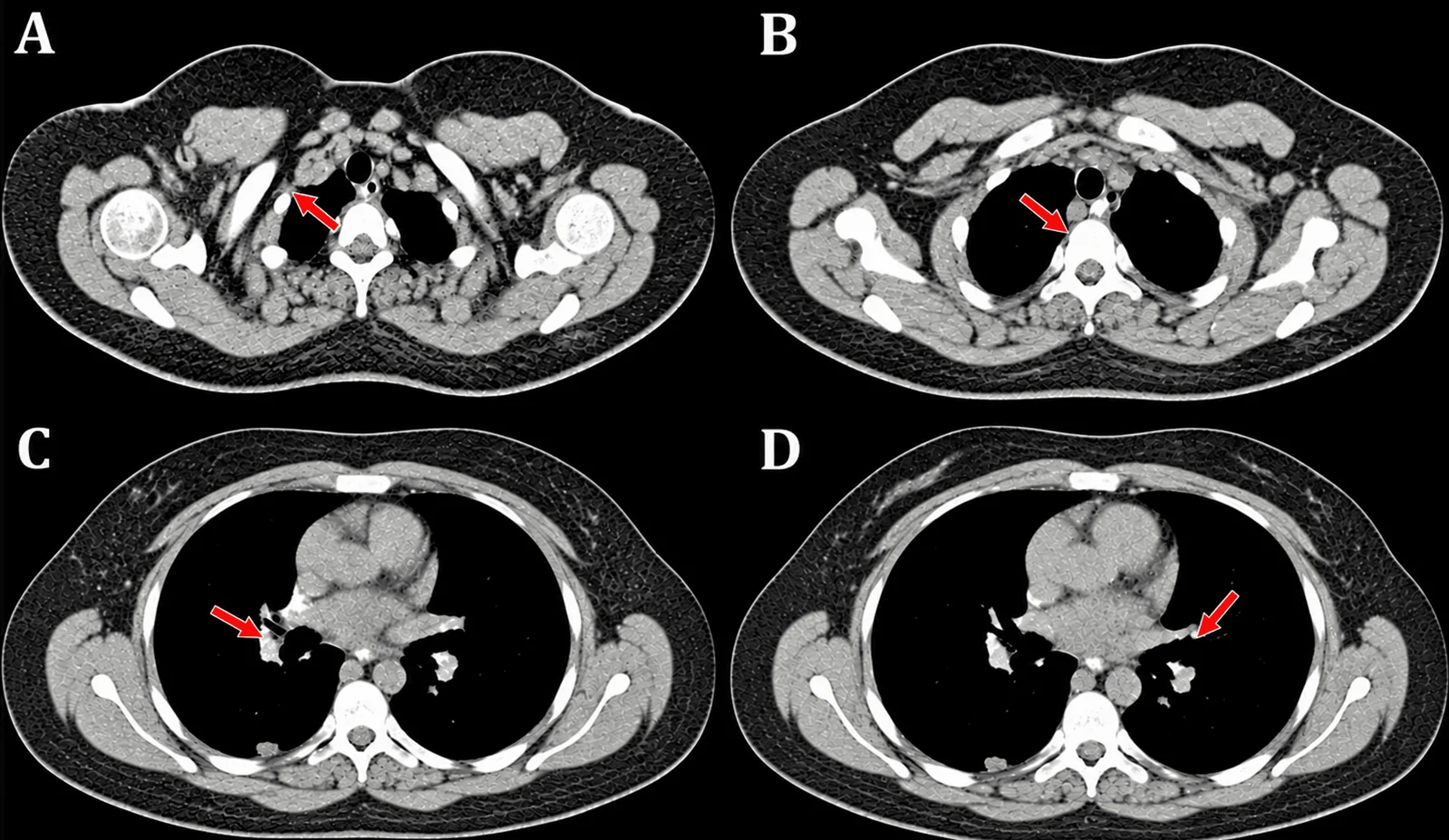
Axial contrast-enhanced computed tomography (CT) images demonstrating pulmonary arterial thromboembolic disease (A–D) Representative axial images obtained at different thoracic levels demonstrating intraluminal filling defects within the pulmonary arterial circulation, involving the right-sided pulmonary arterial branches and left lower-lobar/subsegmental branches. Arrowheads indicate the pulmonary arterial filling defects. The arrowheads were added digitally for annotation and do not alter the original imaging findings.

Further evaluation of the gastric lesion by endoscopic ultrasonography (EUS) in 04/2025 demonstrated a 20.8 × 15.4 mm hypoechoic lesion arising from the fourth sonographic layer of the gastric wall, corresponding to the muscularis propria, in the fundus/body region. The lesion was endophytic and ulcerated, with an appearance considered more suggestive of a gastrointestinal stromal tumor (GIST) than a leiomyoma. EUS-guided biopsy was deferred because of the patient's anticoagulation status. Given the uncertain diagnosis and ulcerated appearance, surgical resection was planned. The decision to defer tissue acquisition was related to the bleeding risk associated with anticoagulation and should not be interpreted as evidence that anticoagulation caused or contributed to the gastric lesion.

In 05/2025, she underwent laparoscopic resection using a three-port approach. The mass was identified on the posterior wall of the gastric fundus near the greater curvature and was excised using a linear stapler. A drain was placed, and the patient recovered without immediate complications. Histopathological examination of the resected specimen demonstrated pancreatic tissue within the gastric wall involving the submucosa and muscularis propria, with well-formed acini, ducts, and islet cells (Figure 3). Focal pancreatitis and reactive inflammatory changes were present, together with chronic active gastritis. Four sampled lymph nodes showed reactive follicular hyperplasia. No malignancy was identified, and the surgical margins were free. The final diagnosis was Heinrich type I gastric pancreatic heterotopia with focal pancreatitis.

**Figure 3:**
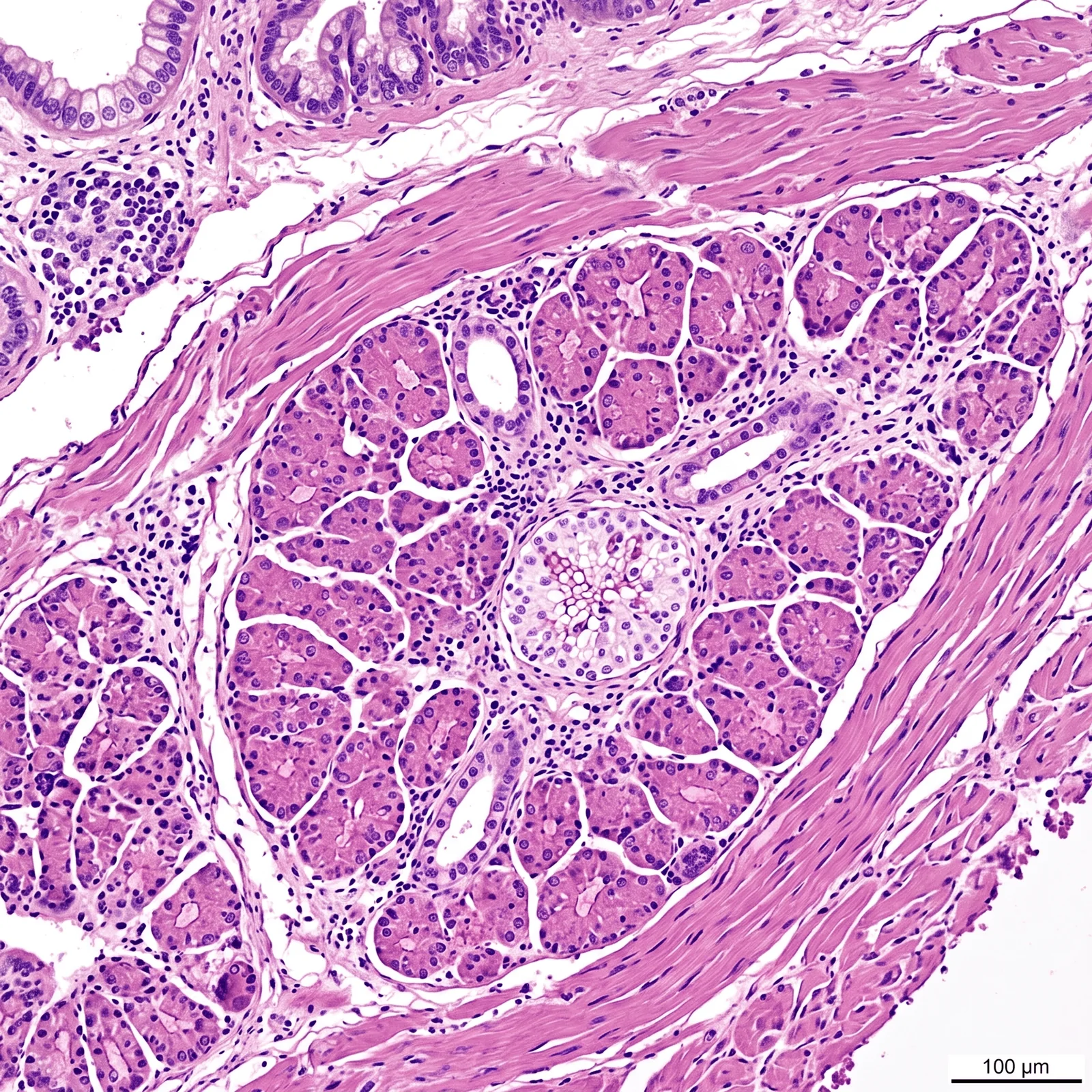
Histopathological findings of gastric pancreatic heterotopia Hematoxylin and eosin–stained section demonstrating well-formed pancreatic acini and ductal structures within the gastric wall, consistent with pancreatic heterotopia. The specimen also shows overlying gastric mucosa and adjacent smooth muscle. Scale bar = 100 μm.

During the subsequent course of her illness, dermatological assessment was performed for multiple hypopigmented, scaly lesions over the extremities. Skin biopsy demonstrated follicular plugging, basement membrane thickening, and superficial perivascular lymphocytic infiltration, consistent with cutaneous lupus erythematosus and favoring discoid lupus erythematosus. The lesions subsequently improved with topical tacrolimus 0.1%.

Perioperative anticoagulation required careful management because of the competing risks of thrombosis and bleeding. Warfarin was withheld perioperatively, with enoxaparin used for bridging, and warfarin was subsequently resumed at 5 mg daily. A subsequent INR of 1.0 was below the intended therapeutic range, and the documented plan was to increase warfarin to 7 mg daily with a target INR of 2.0-3.0. The anticoagulation strategy was individualized to balance the recent pulmonary embolism and thrombotic risk associated with the documented APS diagnosis against the perioperative bleeding risk. The patient continued multidisciplinary follow-up for SLE, APS, and pulmonary embolism. During 2026 follow-up, pulmonary embolism-related changes and bilateral basal triangular-shaped pulmonary consolidations remained evident, while CBC and renal function were reported as within normal limits. In 06/2026, she underwent preconception counseling, with a plan to transition from warfarin to therapeutic enoxaparin before conception. No recurrent hematemesis or immediate postoperative complication was documented in the available follow-up records. The available records also did not provide definitive follow-up imaging confirming resolution or persistence of the bronchoesophageal fistula or documenting recurrence of the gastric lesion; therefore, no conclusion regarding fistula resolution or recurrence can be made.

## Discussion

GPH is an uncommon developmental anomaly that is usually asymptomatic but may produce clinically significant complications. Autopsy studies have reported ectopic pancreatic tissue in approximately 0.5-13.7% of individuals, with the stomach among the most frequent sites [8]. In a systematic review of 934 symptomatic patients, abdominal pain was the most frequent presentation (67%), followed by pancreatitis (28%), gastrointestinal bleeding (9%), and gastric outlet obstruction (9%). Overall, 90% of patients underwent surgical or endoscopic management, while 85% of patients with available follow-up data experienced symptom resolution and 9.7% experienced improvement [2]. These findings demonstrate that although GPH is generally benign, it can produce clinically important inflammatory, ulcerative, hemorrhagic, and obstructive complications.

In the present case, the gastric lesion was initially interpreted as a mesenchymal subepithelial tumor. EUS demonstrated a 20.8 × 15.4 mm hypoechoic lesion arising from the fourth sonographic layer, corresponding to the muscularis propria, with ulceration and an appearance favoring GIST over leiomyoma. This diagnostic difficulty is well recognized. In a clinicopathological study of 184 histologically proven cases, 54.9% of lesions were misdiagnosed preoperatively, while only 14.1% were correctly diagnosed as heterotopic pancreas [9]. Similarly, a study of 31 histologically proven gastric ectopic pancreas lesions found that 53% originated from the fourth EUS layer, with hypoechoic and homogeneous appearances that could closely resemble GIST [10]. A separate study of 31 pathologically confirmed lesions reported a mean size of 10.6 mm, with heterogeneous echogenicity, indistinct borders, and mixed-layer involvement commonly observed [11]. Thus, EUS findings may overlap substantially with other gastric subepithelial tumors, and histopathological confirmation may be required when the diagnosis remains uncertain.

The unusual location and morphology further complicated the preoperative diagnosis in our patient. Gastric heterotopic pancreas is most frequently located in the antrum, whereas the lesion in this case was located in the fundus/body and appeared ulcerated [8,11]. EUS-guided tissue acquisition can assist in establishing the diagnosis in selected patients, although its use depends on lesion characteristics and procedural risk [10,12]. In our patient, biopsy was deferred because of therapeutic anticoagulation, and surgical excision provided definitive diagnosis and treatment. Histopathology demonstrated Heinrich type I pancreatic heterotopia, containing acini, ducts, and islet cells, together with focal pancreatitis and chronic active gastritis. Type I heterotopia contains all major pancreatic components and can therefore develop inflammatory changes similar to those occurring in the orthotopic pancreas [13].

The coexistence of GPH with BEF represents the most unusual aspect of this case. Adult benign, non-iatrogenic BEF is rare and may present with nonspecific respiratory or gastrointestinal symptoms. A systematic review identified 251 individual cases from 183 publications, with congenital malformations accounting for 38.7%, infections for 32.7%, idiopathic fistulae for 13.1%, and other causes for 15.5%. Esophagography demonstrated the highest reported diagnostic sensitivity (97.4%), compared with esophagoscopy (78.9%), CT (49.6%), and bronchoscopy (46.0%). Definitive treatment was surgical in 70% of cases [4]. In our patient, the fistula was initially suspected during EGD because of an abnormal mid-esophageal opening with air and mucus passage and was subsequently demonstrated by CT with oral contrast, which showed communication with the left main bronchus.

Importantly, the association between pancreatic heterotopia and bronchial fistulization has been reported previously. Mohlala and le Roux described a 1989 case of gastrobronchial fistula associated with pancreatic heterotopia involving the gastric fundus [5]. Therefore, the present case should not be described as the first reported association. Its distinctive clinical context is the coexistence of type I GPH, BEF, SLE, APS, recent pulmonary embolism, and therapeutic anticoagulation in a young patient. This combination creates an important diagnostic challenge because the simultaneous presence of an ulcerated gastric subepithelial lesion and an unexpected esophageal-airway communication may suggest a relationship between the two abnormalities, although such a relationship cannot be assumed from coexistence alone.

The mechanism connecting the two abnormalities remains uncertain. Pancreatic heterotopia can develop pancreatitis and local inflammatory changes, and pancreatic secretions could theoretically contribute to adjacent tissue injury [13]. However, the available findings do not establish a direct anatomical or causal relationship between the gastric heterotopia and the BEF. The fistula was identified at the mid-esophageal level near the carina, whereas the gastric lesion was located in the fundus/body. This anatomical separation argues against a demonstrated direct extension from the gastric lesion to the fistula. Therefore, any proposed mechanism involving inflammatory or enzymatic erosion should be considered hypothesis-generating rather than established. The previous report by Mohlala and le Roux provides precedent for the association but does not establish the mechanism in the present patient [5]. Alternative explanations for the BEF, including congenital, inflammatory, infectious, or idiopathic mechanisms, cannot be excluded on the basis of the available clinical information [4,5]. Accordingly, the coexistence of GPH and BEF in this case should be interpreted as an unusual association rather than evidence of causation.

Another important consideration was the interpretation of hematemesis in a patient with SLE and APS receiving anticoagulation. The patient had a recent pulmonary embolism and therefore required anticoagulant therapy, creating a difficult balance between recurrent thrombosis and bleeding. The initial EGD did not identify an active gastric or duodenal bleeding source. Instead, identification of the abnormal esophageal opening prompted further investigation and ultimately established the BEF. The source of the hematemesis therefore remained undetermined. Although the ulcerated GPH, BEF, anticoagulation, or another mucosal process could each be considered in the differential diagnosis, the available findings do not establish any of these as the definitive source of bleeding. This emphasizes that gastrointestinal bleeding in an anticoagulated patient should not automatically be attributed to anticoagulant-associated mucosal injury when an unusual structural abnormality is identified. Conversely, the presence of anticoagulation should not be interpreted as evidence that it caused or contributed to the development of either the gastric lesion or the BEF.

The need for anticoagulation also influenced the diagnostic and perioperative strategy. EULAR recommendations support vitamin K antagonist therapy with a target INR of 2-3 for patients with definite APS and a first venous thrombotic event [14]. Recent observational data in thrombotic APS involving 172 patients and 282 anticoagulation interruptions reported LMWH bridging in 84.7% of warfarin interruptions, with perioperative management tailored to individual thrombotic and procedural bleeding risks [6]. In our patient, warfarin was temporarily withheld, and therapeutic enoxaparin was used for bridging before surgery, followed by resumption of warfarin. This approach reflected the competing risks associated with her recent pulmonary embolism and gastrointestinal bleeding. The anticoagulation strategy was individualized to her recent thromboembolic event and the need for gastric surgery in the setting of active bleeding concerns. The documented postoperative INR of 1.0 prompted a plan to increase the warfarin dose to 7 mg daily, with a target INR of 2.0-3.0. Because the diagnosis of APS was documented clinically and repeat antiphospholipid antibody testing was not available for review, the APS-related laboratory evidence should be interpreted cautiously rather than used to infer persistent antibody positivity.

Finally, resection was reasonable given the lesion's ulcerated appearance, uncertain preoperative diagnosis, and inability to obtain tissue safely while anticoagulated. Surgical or endoscopic treatment is commonly used for symptomatic GPH, although the optimal approach depends on lesion size, depth, symptoms, and diagnostic certainty [2,12,13]. Malignant transformation is exceptionally rare, but a systematic review identified 54 well-documented cases, with the stomach being the most common site and most tumors arising within Heinrich type I heterotopia [15]. In our patient, however, histopathology showed no malignancy and confirmed free surgical margins. The absence of documented postoperative complications and recurrent hematemesis in the available follow-up is reassuring; however, follow-up records did not provide definitive imaging evidence of resolution or persistence of the BEF, and therefore no conclusion regarding fistula resolution or recurrence can be drawn. Similarly, there was no documented evidence of recurrence of the gastric lesion.

## Conclusions

GPH may mimic a gastric subepithelial neoplasm and occasionally present with clinically significant complications. In this patient with SLE, APS, recent pulmonary embolism, and anticoagulation, the coexistence of hematemesis, a gastric lesion, and bronchoesophageal fistula created substantial diagnostic and therapeutic challenges. Multimodal imaging and histopathology were essential for establishing the final diagnosis of Heinrich type I GPH with focal pancreatitis. Careful perioperative anticoagulation management was also required because of the competing risks of bleeding and recurrent thrombosis. Importantly, although GPH and bronchoesophageal fistula coexisted in this patient, no direct anatomical or causal relationship between the two abnormalities could be demonstrated, and the source of hematemesis remained undetermined. This case highlights the importance of maintaining a broad differential diagnosis when an unusual gastric subepithelial lesion is identified alongside an esophageal-airway communication, particularly in patients with competing thrombotic and bleeding risks.
